# Control of positive end-expiratory pressure (PEEP) for small animal ventilators

**DOI:** 10.1186/1475-925X-9-36

**Published:** 2010-07-30

**Authors:** Antonio Giannella-Neto, Gabriel C da Motta Ribeiro, Edil L Santos, João HN Soares, Marcelo V Leão Nunes, Frederico C Jandre

**Affiliations:** 1 Pulmonary Engineering Laboratory, Biomedical Engineering Program, COPPE-Federal University of Rio de Janeiro, RJ, Brazil

## Abstract

**Background:**

The positive end-expiratory pressure (PEEP) for the mechanical ventilation of small animals is frequently obtained with water seals or by using ventilators developed for human use. An alternative mechanism is the use of an on-off expiratory valve closing at the moment when the alveolar pressure is equal to the target PEEP. In this paper, a novel PEEP controller (PEEP-new) and the PEEP system of a commercial small-animal ventilator, both based on switching an on-off valve, are evaluated.

**Methods:**

The proposed PEEP controller is a discrete integrator monitoring the error between the target PEEP and the airways opening pressure prior to the onset of an inspiratory cycle. In vitro as well as in vivo experiments with rats were carried out and the PEEP accuracy, settling time and under/overshoot were considered as a measure of performance.

**Results:**

The commercial PEEP controller did not pass the tests since it ignores the airways resistive pressure drop, resulting in a PEEP 5 cmH_2_O greater than the target in most conditions. The PEEP-new presented steady-state errors smaller than 0.5 cmH_2_O, with settling times below 10 s and under/overshoot smaller than 2 cmH_2_O.

**Conclusion:**

The PEEP-new presented acceptable performance, considering accuracy and temporal response. This novel PEEP generator may prove useful in many applications for small animal ventilators.

## Background

The choice of the adequate positive end-expiratory pressure (PEEP) is one of the main concerns about ventilatory settings for mechanical ventilation. From normal lung subjects during anesthesia to patients with acute lung injuries (ALI), the use of a PEEP equal to zero is practically absent in the current evidence based ventilatory therapy [1,2].

In commercial microcontrolled artificial ventilators for humans, the up-to-date technology for PEEP control is most commonly done by an expiratory valve with a membrane that imposes a counter pressure regulated by an electromechanical device. At the beginning of expiration, the pulmonary pressure being higher than the PEEP enables the valve to open and expiration remains until equalization of both pressures or until the expiration ceases because of the start of the next inspiration.

An alternative approach is to eliminate the membrane valve by using an on-off valve to set the target PEEP. In this case, the valve is kept open to the atmosphere when expiration begins and closes when the target PEEP is achieved. Some potential benefits may be identified with this technique. First, the driving pressure for expiration is magnified, and consequently the expiratory time may be diminished [3,4]. Second, the elimination of a membrane circumvents common adversities found in practice, mainly the mechanical oscillation of the membrane and the airflow resistance imposed by it. Additionally, an on-off valve can be easily miniaturized for small animal setups, in which minimal compressive volume of the respiratory circuit is mandatory.

The objective of this work is the evaluation of two PEEP control systems based on switching an on-off valve installed on the expiratory circuit. The first is the PEEP of a commercial ventilator and the second is a prototype developed by the authors. Both systems are based on the same principle, i.e., to close the on-off valve at a certain point of expiration, in order to achieve the target PEEP for the rest of the expiratory time. The performance of both systems for controlling the PEEP was tested in vitro and in vivo in a rat model.

## Methods

The commercial ventilator was an INSPIRA model 557059 (Harvard Apparatus, MA, USA). The information available on the website of the manufacturer is that the PEEP feature "allows a positive pressure to be maintained between inspirations instead of falling to zero or near zero at the end of the expiratory phase. When the PEEP pressure is reached, the expiration valve closes until the next inspiration cycle begins." The PEEP generated by this ventilator was denominated PEEP-old. A specimen of the INSPIRA ventilator ASVP, serial number B-45397 (Harvard Apparatus, MA, USA) regularly purchased has been used in all tests.

In this work, a prototype PEEP controller was designed, to operate in conjunction with the ventilator INSPIRA. The system includes a miniaturized on-off valve 003-0459-900 (Parker, OH, USA) connected to the exhaust port of the ventilator. To control such valve, two signals, available from the ventilator, were employed: the airways opening pressure (P_ao-Inspira_) and a binary signal of synchronism (S_I-E_), in which the logical one indicates the occurrence of the inspiratory cycle and the logical zero, the expiratory cycle. These two signals were acquired at a sampling rate of 1000 Hz by an analog-to-digital (A/D) converter PCM-3718HG (Advantech, CA, US) installed on a personal computer PCM-6898 (AAEON Electronics, NJ, US) running a Simulink model using the Real-Time Windows Target (Mathworks, US). The controller reads P_ao-Inspira _and S_I-E_, and computes the duration of the opening of the on-off valve during the expiration, τ_exp_. From S_I-E_, for the n-th respiratory cycle the controller calculates the total duration of the expiration as reported by the ventilator, T_exp_(n). Then, from P_ao-Inspira _and the target PEEP (PEEP_T_), the controller updates τ_exp _for the n-th respiratory cycle by the law:

where PEEP_I _is the intrinsic PEEP [3], measured immediately before the beginning of the n-th inspiration while the on-off valve is still closed, and g(PEEP_T_) has values that depend on PEEP_T_, equal to 0.08 (0 ≤ PEEP_T _≤ 3 cmH2O), 0.03 (3 < PEEP_T _≤ 5 cmH2O), 0.01 (5 < PEEP_T _≤ 10 cmH2O) and 0.006 (PEEP_T _> 10 cmH2O). The values of g were adjusted empirically by numerical simulation in order to reduce the settling time as well as the under/overshoot of the response. The values of τ_exp _were limited by software from T_exp_/12 to T_exp_. The controller outputs the signal to a driver circuit, switched by a digital output of the A/D card. The PEEP generated by this system is hereafter called PEEP-new.

During tests, the signals of interest were also continuously monitored and digitized at a sampling rate of 1000 Hz by an A/D converter 6008 (National Instruments, TX, US) and stored in a personal computer running a program written in LabVIEW (National Instruments, TX, US).

### Performance Tests

In order to test the performance characteristics of the PEEP control system, the following definitions have been applied: for each respiratory cycle, the PEEP was calculated as the mean value of the airways opening pressure for the last 10 ms of the expiratory phase; during a PEEP step change, the steady-state PEEP was calculated as the mean value of the last 20 respiratory cycles of a period with constant PEEP; the settling time was determined as the time after which the difference between the actual PEEP and the steady-state PEEP was less than ± 0.5 cmH_2_O; the overshoot (undershoot) was found as the highest PEEP deviation from the steady-state PEEP after the rise (fall) time, considered as the period of time of PEEP increasing (decreasing) from the start of a PEEP step change up to the first cross with the new target PEEP.

### In vitro tests

**Figure 1:**
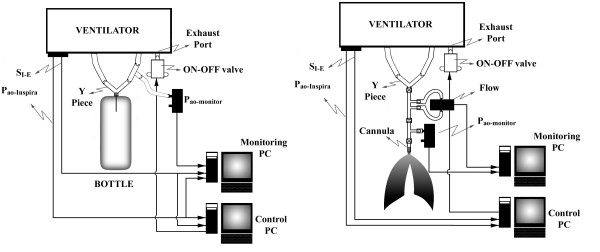
**Experimental set-up**. Experimental setups. Left panel: in vitro **- **a physical model was employed during experiments; Right panel: in vivo **- **here the respiratory flow was monitored. S_I-E _= logic signal that identifies the inspiratory as well as the expiratory cycles; P_ao _= airways opening pressure; The ON-OFF valve is the valve employed to control the PEEP.

Figure 1-a shows the experimental set-up. A physical model of the respiratory system (RS) of a rat has been used for tests. It consisted of a bottle of 500 ml, whose compliance, around 0.5 ml/cmH_2_O, is within the range of the compliance of a healthy RS of a rat [5]. A Y piece for rats 73-2846 (Harvard Apparatus, Ma, US) was inserted into the compliance model. An additional resistor, representing the airways resistance (R_aw_), was not included in the model since the cannula of the Y piece presented a resistance in the order of magnitude of the airways resistance of a rat with healthy lungs [6]. The P_ao _(P_ao-monitor_) was monitored with a pressure transducer 163PC01D48 (Honeywell, NJ, US) connected to a T piece placed in the expiratory limb, close to the Y piece. In the instances when the PEEP-old was evaluated, tubes and connections were employed as suggested by the manufacturer. When the PEEP-new was tested, the set-up was the same with the inclusion of the on-off valve. The total expiratory circuit resistance from the cannula to the atmosphere including all connecting tubes, the ventilator's on-off valve and the additional on-off valve for PEEP-new control system was of 390 cmH2O.l^-1^.s^-1^. The additional on-off valve represented about 50% of the total expiratory circuit resistance.

Both PEEP generators were tested with the same respiratory settings. The ventilator was set in volume control mode with a tidal volume (V_T_) of 3 ml, a respiratory frequency (RF) of 60 breaths per minute, an inspiratory to expiratory time ratio (I:E) of 1:1, and ventilated with ambient air.

The PEEP-old was tested for the targets of 3, 5 and 10 cmH_2_O, in increasing as well as decreasing steps. The trials were triplicate. Since the results with PEEP-old showed large deviations from the target PEEP (more than 5 cmH_2_O), the following test was performed only for the PEEP-new method. Initially, the PEEP was set to zero cmH_2_O, and sequentially it was changed to 3, 5, 10, 15, 10, 5, 3 and again zero cmH_2_O. The duration of each PEEP step was of 1 min, controlled by the computer. Again, the trials were repeated three times.

### In vivo tests

Figure 1-b shows the in vivo experimental setup. After the results of the in vitro experiments in which the resistances of the Y plus the cannula's revealed to be fairly obstructive (see Results), another, less resistive Y connector was employed. To the common limb of the Y a unicapillary pneumotachometer, designed and calibrated according to Giannella-Neto et al. [7], was connected, together with a small tube with a lateral port for the measurement of P_ao-monitor _calibrated against a reference instrument Timeter RT-200 (Allied HealthCare Products, Mo, US). A short, low resistive cannula (ID of 1.5 mm, 30 mm long) was placed to fit to the trachea. The flow rate (), P_ao-monitor _and ECG were continuously monitored, digitized at 1000 Hz each and stored on hard-disk.

As an in vivo pilot experiment, three Sprague-Dawley rats weighting 220 ± 15 g were mechanically ventilated in a protocol approved by the local Ethical Committee. The animals were sedated, anesthetized and paralyzed. The respiratory settings were the same as the in vitro experiments.

Initially, a baseline condition with PEEP-new of 3 cmH_2_O was performed. The PEEP-old was tested three times at the levels of 3 and 5 cmH_2_O. Similarly as the in vitro experiments, it failed to follow the target PEEP. The subsequent protocol was applied only for the PEEP-new, which consisted in decreasing then increasing PEEP in 1-minute steps of 1 cmH_2_O, starting and ending at a pressure of 9 cmH_2_O.

## Results

### In vitro

**Figure 2:**
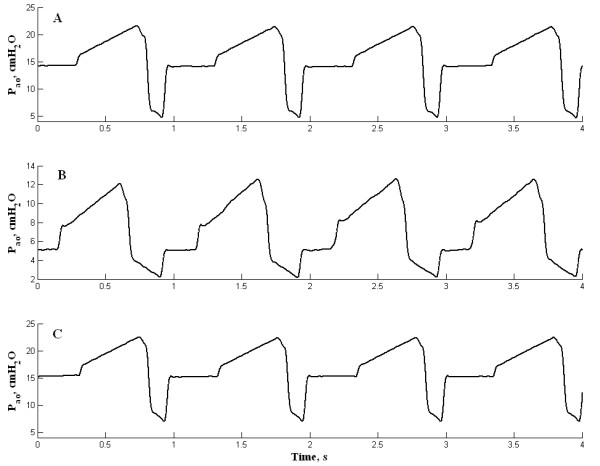
**Airways opening pressure waveforms**. a) The airways opening pressure (P_ao_) for a target PEEP of 5 cmH_2_O controlled by PEEP-old method; b) and c) The P_ao _for the targets PEEP of 5 and 15 cmH_2_O, respectively, controlled by PEEP-new method. Note that for a target PEEP of 5 cmH_2_O the PEEP-new closes at a P_ao _around 2 cmH_2_O.

Figure (2a and 2b) shows the waveforms of the P_ao-Inspira _for both PEEP methods during the in vitro tests, at a PEEP of 5 cmH_2_O. The PEEP-old resulted in around 14 cmH_2_O, whereas the PEEP-new was very close to the set point. For comparison, Figure 2-c shows the P_ao-Inspira _for a PEEP-new of 15 cmH_2_O. It can be noted that the waveform is very similar to that depicted in Figure 2-a. In both cases, the expiration was interrupted at a pressure around 5 cmH_2_O.

The PEEP-old was tested at pressures of 3, 5 and 10 cmH_2_O resulting in 9.0 ± 0.3, 13.9 ± 0.2, and 26.9 ± 0.5 cmH_2_O, respectively. In all cases, the PEEP resulted more than twice the target value.

Table 1 presents the PEEP step change test with PEEP-new. The PEEP control was very accurate since for all trials and PEEP values the maximal deviation from the target was of 0.2 cmH_2_O. Considering all trials, the maximal overshoot/undershoot was smaller than ± 1.3 cmH_2_O and the highest settling time was of about 10 s.

**Table 1:** Performance of PEEP-new during the PEEP step change test in vitro

**PEEP target (cmH**_**2**_**O)**	**PEEP (cmH**_**2**_**O)**			**Overshoot (cmH**_**2**_**O)**			Settling time (s)		
	
	Trial 1	Trial 2	Trial 3	Trial 1	Trial 2	Trial 3	Trial 1	Trial 2	Trial 3
**0**	0.5	0.6	0.5	-	-	-	-	-	-
**3**	3.1	3.1	3.1	0.4	0.6	0.3	4.1	1.0	3.0
**5**	5.1	5.1	5.1	0.3	0.5	0.2	5.1	1.0	4.1
**10**	10.2	10.2	10.2	1.0	1.0	1.0	9.1	7.1	8.1
**15**	15.2	15.2	15.1	1.3	1.2	1.1	10.2	7.1	6.9
**10**	10.0	10.1	10.1	-0.9	-1.0	-1.2	6.1	4.1	5.1
**5**	5.1	5.0	5.1	-0.9	-0.9	-0.9	3.0	2.0	3.0
**3**	3.1	3.3	3.0	-0.5	-0.4	-0.5	2.0	1.0	2.0
**0**	0.5	0.5	0.5	-	-	-	-	-	-

The PEEP step change temporal order follows the table rows. Overshoot and settling time are not reported for the first PEEP value, as well as for PEEP = 0 since at this condition the controller is not operating.

### In vivo

Similarly to the in vitro tests, the PEEP-old was unable to follow the target PEEP. The overall results for all animals for the PEEPs of 3 and 5 cmH_2_O were 6.7 ± 0.6 and 9.8 ± 1.5 cmH_2_O, respectively. The deviations from the target found in vivo revealed to be smaller than in vitro, and this result will be discussed later.

Table 2 presents the in vivo PEEP step change test with PEEP-new. The steady-state PEEP was very close to the target at all PEEP values, and the settling time was bound to about 10 s for all animals. However, in one animal (rat 3) a overshoot/undershoot up to ± 1.8 cmH_2_O was noted in some steps after the PEEP of 2 cmH_2_O during the descending phase.

**Table 2:** Performance of PEEP-new during the PEEP step change test in vivo

**PEEP target (cmH**_**2**_**O)**	**PEEP (cmH**_**2**_**O)**			**Overshoot (cmH**_**2**_**O)**			Settling time (s)		
	
	Rat 1	Rat 2	Rat 3	Rat 1	Rat 2	Rat 3	Rat 1	Rat 2	Rat 3
**9**	9.0	9.0	9.0	-	-	-	-	-	-
**8**	8.0	8.0	8.0	-0.1	-0.1	-0.1	5.1	5.1	6.1
**7**	7.0	7.0	7.0	-0.1	-0.1	-0.2	5.1	6.1	6.1
**6**	6.0	6.0	6.0	-0.1	-0.1	-0.2	6.1	5.1	5.1
**5**	5.0	5.0	5.0	-0.3	-0.2	-0.2	3.1	3.0	2.0
**4**	4.0	4.0	4.0	-0.2	-0.5	-0.2	3.1	3.0	4.1
**3**	3.0	3.0	3.0	-0.4	-0.2	-0.2	2.0	2.0	2.0
**2**	2.0	1.9	2.0	-0.2	-0.2	-1.8	2.0	2.0	2.0
**1**	0.8	0.8	1.0	-0.3	-0.2	-0.6	2.0	3.0	5.1
**0**	0.4	0.3	0.5	-	-	-	-	-	-
**1**	0.8	0.9	1.0	0.1	0.1	1.1	0.0	1.0	6.1
**2**	1.9	1.9	1.9	0.1	0.1	1.0	2.0	2.0	3.0
**3**	3.0	2.9	3.0	0.3	0.2	0.4	2.0	1.0	1.0
**4**	4.0	3.9	4.0	0.2	0.2	0.4	3.1	2.0	2.0
**5**	5.0	5.0	5.0	0.3	0.1	0.3	3.1	2.0	4.1
**6**	6.0	6.0	6.0	0.1	0.1	1.8	6.1	5.1	7.1
**7**	7.0	7.0	7.0	0.1	0.1	0.2	4.1	4.1	10.2
**8**	8.0	8.0	8.0	0.1	0.1	0.5	5.1	4.1	8.1
**9**	9.0	9.0	9.0	0.1	0.2	0.2	4.1	4.1	5.1

The PEEP step change temporal order follows the table rows. Overshoot and settling time are not reported for the first PEEP value, as well as for PEEP = 0 since at this condition the controller is not operating.

**Figure 3:**
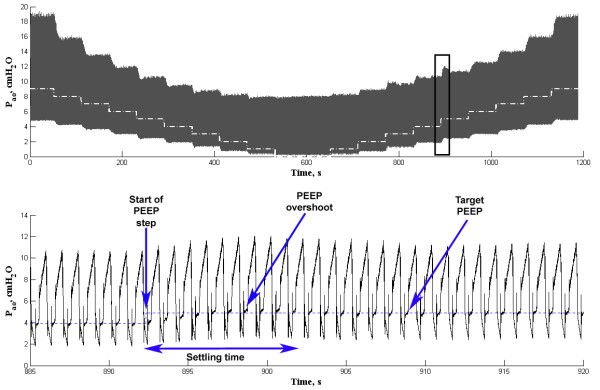
**Airways opening pressure during PEEP step change test**. Upper panel: airways opening pressure (P_ao_) during the entire PEEP step change test with PEEP-new during one in vivo experiment. The dashed white lines are the target PEEP. Lower panel: detail of P_ao _during the PEEP step change from 4 to 5 cmH_2_O. For this step, the arrows indicate the start of the PEEP step, the settling time, the overshoot and the target PEEP.

Figure 3 shows the P_ao _during the PEEP step change test protocol with PEEP-new during one in vivo experiment (animal #1).

## Discussion

The PEEP-old controller did not pass the tests since the PEEP obtained largely deviated from the target PEEP in both in vitro and in vivo tests. Most apparently, its controller responds instantaneously to the pressure, closing the valve when the P_ao _reaches the target PEEP during expiration, and thus the dynamic evaluation tests were not performed. In almost all circumstances, after a PEEP condition was set, the ventilator reported a "high PEEP alarm" indicating that the actual end-expiratory pressure exceeded 11 cmH_2_O or the target PEEP plus 5 cmH_2_O, whichever was less. Since the ventilator did not include a safety valve, the high PEEP condition continued to be present until the operator changed the settings. The P_ao _waveform during the PEEP-old control showed that the valve was switched at the moment when the P_ao _was equal to the target PEEP. Considering that at the switching of the valve P_ao _was equal to the elastic pressure minus the resistive pressure of the respiratory system, and the resistive pressure was zeroed by interrupting the flow, the P_ao _suddenly rose by the same amount (see Figure 2). The in vitro PEEP deviation was higher than in vivo, most probably because the cannula employed in vitro was more resistive than the total resistance in the in vivo experiments, which included a Y-piece plus a flexible catheter and the animal's airways.

The PEEP-new controller presented an adequate performance. The in vitro tests resulted in a maximal PEEP deviation from the target of 0.2 cmH_2_O, maximal overshoot of 1.3 cmH_2_O and maximal settling time of 10.2 s for all trials and all PEEP values (Table 1). The evaluation in vivo showed a similar performance, with the exception of animal #3, in which during the descending phase of the PEEP step change test at PEEP of 2 cmH_2_O, the presence of liquids in the airways resulted in increase of the airways resistance. In consequence, the controller opened the expiratory valve to the atmosphere for a period very close to the expiratory period and for some target PEEP, high overshoot/undershoot occurred during a PEEP step change. Nevertheless, also in these circumstances the controller could adjust the PEEP to the target.

In the present implementation of the PEEP-new method, some features available in the commercial ventilator were used, such as the S_I-E _signal and the P_ao-Inspira_. Nevertheless, the PEEP-new may be developed for a general ventilator, simply employing the set-up for in vivo experiments (Figure 1, right panel) where the P_ao _as well as the flow rate were monitored independently. In such a case, the inspiratory or expiratory phases may be identified by the sign of the flow rate. Additionally, the present controller design did not use any information regarding the mechanics of the RS to control the on-off valve switching moment. Alternatively, Pino and Giannella-Neto [4] showed by numerical simulations a PEEP control technique with the real-time estimation of the RS expiratory time constant, including the breathing circuit and the endotracheal tube, and the control was adequate also for RS including viscoelastic properties.

Setting the PEEP at PEEP_I _was employed by East et al. [3] in pressure-controlled inverse ratio ventilation (PCIRV), to provide high mean airway pressure (Paw) at the lowest peak inspiratory pressure, while maintaining a desired level of PEEP_I_, tidal volume, and arterial pH. In order to maximize the Paw, the lowest expiratory time was chosen without any expiratory pause. In the present work a simple protocol was designed to control the PEEP during volume control mode. We did not control I:E which was fixed in 1. Have the I:E control been also considered, we could, as proposed by East et al. [3], seek to maximize the mean Paw, or conversely, as previously described [4], to minimize the mean Paw by extending the expiratory pause for a chosen PEEP. Clinically, some consequences may be foreseen. East et al. [3] reported hemodynamic effects with a decrease of the cardiac output and an increase in both pulmonary artery and right atrium pressures. It may be hypothesized that the hemodynamic effects of the minimization of the mean Paw would be the opposite. A lower mean alveolar pressure is potentially favorable to minimize the effects of PEEP on the pulmonary vascular resistance by preventing dynamic hyperinflation [8]. However, a ventilatory effect in the lungs could be considered as well, since the decrease of the mean Paw could reduce recruitment of alveolar units, with the impairment of gas exchange and increase of venous admixture. These speculations deserve a clinical evaluation. Technically, despite the algorithm of East et al. [3] being more complex since it considers more controls than ours, in what concerns specifically the PEEP controller both methods are based on a similar integral controller in which the updating of the effective expiratory period depends on the difference [PEEP_I _- PEEP_T_].

Some limitations of this study must be reported. The present controller, as it is, cannot be used with spontaneously breathing subjects. Furthermore, the present controller was tuned for the mechanical ventilation of rats. In consequence, the reported performance indices cannot be generalized to other species. The controller law parameters may have to be retuned for use with different respiratory mechanics. The hardware presented a respiratory circuit resistance which was appropriate for ventilating rats and mice; however for larger animals, the on-off valve must be less resistive.

The use of small animals for studies related to the mechanical ventilation settings and its effects on ventilator-induced lung injuries and inflammatory responses is very frequent. Some commercial ventilators for humans may be suitable to typical respiratory rates and tidal volumes for rats [9], albeit the breathing circuit must be adapted in order to minimize the compressed volume. For mouse models, however, a small animal ventilator is mandatory since the V_T _may be smaller than 200 μl. The PEEP-new may represent an alternative for an accurate PEEP implementation capable of being computer controlled, as in the time-programmed PEEP step change test shown in Figure 3. The availability of the flow rate and P_ao _allows estimating the parameters of respiratory mechanics and, furthermore, the entire system can be programmed to automatically control the PEEP as performed by Jandre and coworkers [10].

## Conclusions

The PEEP-old controller resulted in PEEP values always higher than the target, in both in vitro and in vivo tests. The PEEP-new controller presented acceptable performance, considering accuracy and temporal response. This novel PEEP generator may be implemented at reasonable costs and may prove useful in many applications for small animal ventilators.

## Competing interests

The authors declare that they have no competing interests.

## Author's contributions

AGN and FCJ designed the study and wrote the paper. CGMR and FCJ developed the controller. AGN, GCMR, MVLN, JHS participated in the experiments. AGN, GCMR and ELS analyzed the results.

All authors have read and approved the final manuscript.
